# Upconversion Nanoparticles-Encoded Hydrogel Microbeads-Based Multiplexed Protein Detection

**DOI:** 10.1007/s40820-017-0184-y

**Published:** 2017-12-29

**Authors:** Swati Shikha, Xiang Zheng, Yong Zhang

**Affiliations:** 1 Department of Biomedical Engineering, Faculty of Engineering, National University of Singapore (NUS), 4 Engineering Drive 3, Block E4 #04-08, Singapore, 117583 Singapore; 2 NUS Graduate School for Integrative Sciences and Engineering, Centre for Life Sciences (CeLS), 05-01 28 Medical Drive, Singapore, 117456 Singapore

**Keywords:** Upconversion nanoparticles, PEGDA microbeads, Encoding, Multiplexed bio-detection, Single wavelength excitation

## Abstract

Fluorescently encoded microbeads are in demand for multiplexed applications in different fields. Compared to organic dye-based commercially available Luminex’s xMAP technology, upconversion nanoparticles (UCNPs) are better alternatives due to their large anti-Stokes shift, photostability, nil background, and single wavelength excitation. Here, we developed a new multiplexed detection system using UCNPs for encoding poly(ethylene glycol) diacrylate (PEGDA) microbeads as well as for labeling reporter antibody. However, to prepare UCNPs-encoded microbeads, currently used swelling-based encapsulation leads to non-uniformity, which is undesirable for fluorescence-based multiplexing. Hence, we utilized droplet microfluidics to obtain encoded microbeads of uniform size, shape, and UCNPs distribution inside. Additionally, PEGDA microbeads lack functionality for probe antibodies conjugation on their surface. Methods to functionalize the surface of PEGDA microbeads (acrylic acid incorporation, polydopamine coating) reported thus far quench the fluorescence of UCNPs. Here, PEGDA microbeads surface was coated with silica followed by carboxyl modification without compromising the fluorescence intensity of UCNPs. In this study, droplet microfluidics-assisted UCNPs-encoded microbeads of uniform shape, size, and fluorescence were prepared. Multiple color codes were generated by mixing UCNPs emitting red and green colors at different ratios prior to encapsulation. UCNPs emitting blue color were used to label the reporter antibody. Probe antibodies were covalently immobilized on red UCNPs-encoded microbeads for specific capture of human serum albumin (HSA) as a model protein. The system was also demonstrated for multiplexed detection of both human C-reactive protein (hCRP) and HSA protein by immobilizing anti-hCRP antibodies on green UCNPs.

**Electronic supplementary material:**

The online version of this article (10.1007/s40820-017-0184-y) contains supplementary material, which is available to authorized users.

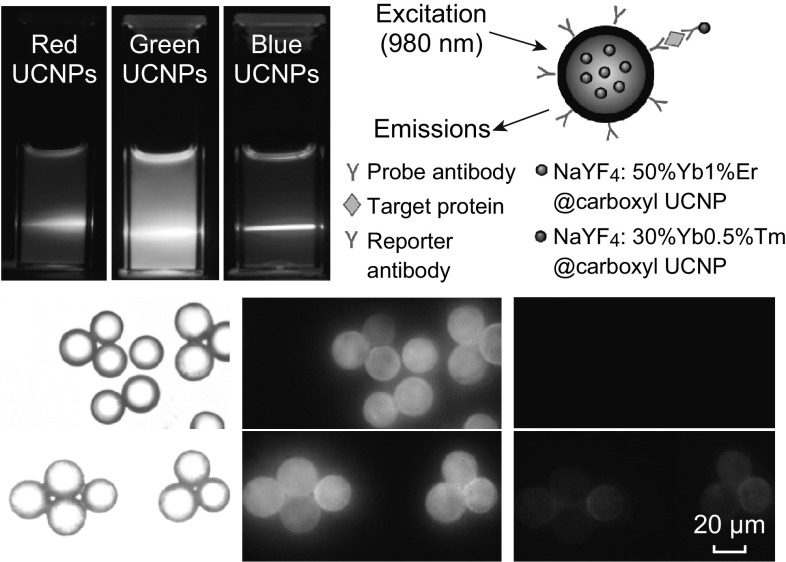

## Highlights

- Upconversion nanoparticles (UCNPs) were used for encoding as well as labeling reporter antibody for multiplexed detection.
- UCNPs-encoded microbeads with uniformity in shape, size and fluorescence intensity were prepared by droplet microfluidics method.
- Surface modification of poly(ethylene glycol) diacrylate microbeads achieved by silica coating followed with carboxyl modification.

## Introduction

Multiplexed bio-detection allows simultaneous detection of multiple biomolecules in a single run and is important for applications in different fields such as high-throughput drug screening [1–3], genotyping [4–6], proteomics [7–9], disease diagnosis [10], and environmental engineering [11, 12]. It can be performed in two different formats: traditional planar arrays [13–15] or beads-based bioassays [16, 17]. Compared to planar arrays, bead-based bioassays are endowed with advantages such as large surface area-to-volume ratio, efficient binding kinetics, higher flexibility to perform a wide range of tests, easy preparation and handling of samples [18, 19]. To perform the multiplex bio-detection, the beads are normally encoded with unique signatures for easy identification of analyte bound to the specific bead that targets it [20, 21]. Out of the available methods of encoding, fluorescence-based optical encoding is most favored owing to their ease of identification, high sensitivity, and signal localization [21, 22]. Commercially established and widely used Luminex’s xMAP technology uses red and orange dyes at different ratios to encode the beads and a green dye for detection of targets [23]. However, it suffers from certain limitations such as those associated with the inherent property of the organic dyes that includes photobleaching, spectral overlapping, need for multiple excitation sources, and autofluorescence [21]. With regard to this, UCNPs having advantages such as photostability, large anti-Stokes shift, nil background autofluorescence, low photodamage, and single wavelength excitation are better alternatives to currently used organic dyes [24–28]. Henceforth, this study aims to use UCNPs for both encoding of beads as well as labeling of reporter antibody.

Beads encoding can be done by ex situ and in situ strategies, with nanoparticles either encapsulated inside the beads or decorated on the surface. In ex situ methods, both the nanoparticles and beads are preformed prior to the encoding process. However, in in situ strategies, either the beads or nanoparticles are simultaneously formed in situ during the encoding process. Currently, UCNPs-encoded beads are prepared by swelling-based ex situ method. In this strategy, porous beads are dispersed in a swelling solvent containing UCNPs. Based on the concentration gradient, these UCNPs diffuse into the porous beads. Upon removal of the swelling solvent, the beads shrink and retain the UCNPs inside it [16]. However, UCNPs-encoded beads prepared by this strategy were found to have non-uniform distribution of UCNPs inside the beads. Moreover, some of the UCNPs were confined on the beads surface, thereby hampering surface modification of these beads. Additionally, the swelling strategy is rather laborious and, along with the above-mentioned drawbacks, is considered unfavorable for use in high-throughput synthesis of UCNPs-encoded beads. Another strategy was reported to use stop-flow lithography producing UCNPs-encoded microbars [29]. However, the process was complicated, and the size of encoded microbars (250 × 70 μm^2^) is too large to perform bead-based assays. Bearing these in mind, droplet microfluidics was adopted in this study to prepare uniformly distributed UCNPs-encoded microbeads in a high-throughput manner.

Droplet microfluidics has been reported to generate uniformly encoded microbeads with controlled size as well as distribution of the encapsulated fluorescent particles inside the microbeads, in a high-throughput manner [30, 31]. To prepare the beads, UCNPs dispersed in water containing the bead precursors is co-flown with an immiscible oil phase to produce a hydrogel that is further solidified by photocrosslinking in the presence of a photoinitiator.

**Fig. 1:**
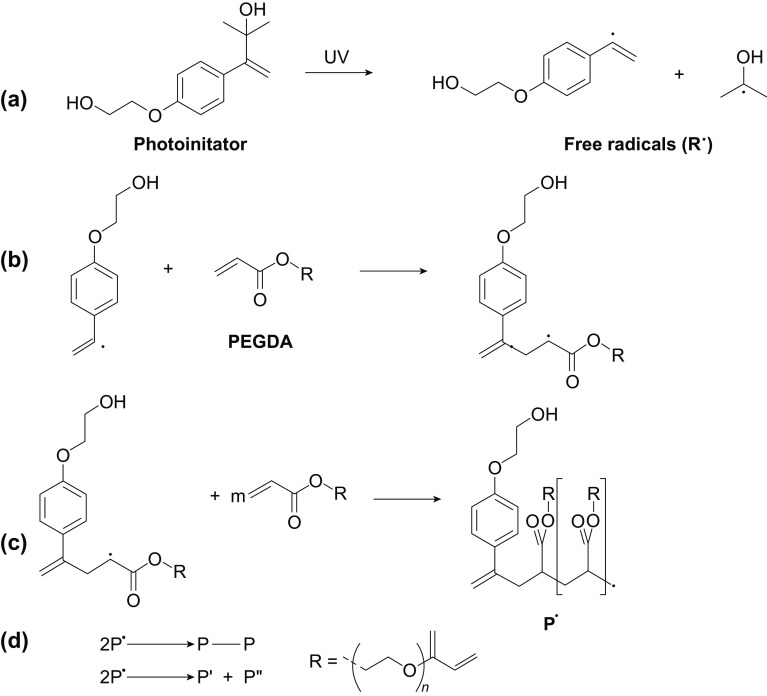
Photocrosslinking mechanism of PEGDA. **a** UV-induced dissociation of photoinitiator to generate free radicals. **b** Propagation step, where free radical attacks the double bond in PEGDA to form new radicals. **c** Chain transfer. **d** Different combinations of free radicals to form stable, inactive products, thereby leading to chain termination [1]

Here, poly(ethylene glycol) diacrylate (PEGDA) microbeads were prepared by the process of photopolymerization in the presence of 2-hydroxy-4′-(2-hydroxyethoxy)-2-methylpropiophenone, a UV-sensitive photoinitiator. The process of photopolymerization consists of several steps including initiation, propagation, chain transfer, and termination. As shown in Fig. 1, upon exposure to UV light, the photoinitiator dissociates to yield free radicals that attack the double bond present in PEGDA to trigger the propagation reaction. Eventually, when the source polymers are exhausted in the mixture, the reaction terminates, and a stable crosslinked product is obtained [32]. NaYF_4_:50%Yb1%Er and NaYF_4_:18%Yb2%Er30%Lu UCNPs emitting red and green colors, respectively, were prepared and surface modified for uniform distribution inside the microbeads after encapsulation. Multicolor microbeads were prepared by mixing these two colors of UCNPs at different ratios before encapsulating them inside the microbeads using droplet microfluidics. The UCNPs-encoded microbeads were surface modified with carboxyl groups to impart functionality for conjugation of probe antibodies against target proteins. The detection was performed by sandwich immunoassay using reporter antibody labeled with NaYF_4_:30%Yb0.5%Tm UCNPs emitting blue color. Lastly, multiplexed bio-detection was demonstrated using human serum albumin (HSA) and human C-reactive protein (CRP) as model target proteins.

## Materials and Methods

### Materials

To prepare the photomask with droplet generator design, silicon wafers were purchased from Teltec (Singapore), SU-8 2050 (photoresist) and SU-8 developer were purchased from Microchem (Newton, MA), and Sylgard-184 (Poly(dimethylsiloxane), PDMS) was purchased from Dow Corning (Midlance, MI). HFE-7500 was purchased from 3M Company and used without further purification. Surfactant Pico-surf™ 2 were obtained from Sphere Fluidics Ltd. (Cambridge, UK) and used without further purification. PEGDA (bead precursor, average M_n_ 575), 2-hydroxy-4′-(2-hydroxyethoxy)-2-methylpropiophenone (photoinitiator), yttrium chloride (YCl_3_·6H_2_O, 99.9%), ytterbium chloride (YbCl_3_·6H_2_O, 99.9%), erbium chloride (ErCl_3_·6H_2_O, 99.9%), lutetium chloride (LuCl_3_·6H_2_O, 99.9%), thulium chloride (TmCl_3_·6H_2_O, 99.9%), sodium hydroxide (NaOH, 98+%), ammonium fluoride (NH_4_F, 98+%), 1-octadecence (90%), oleic acid (OA, 90%), and tetraethyl orthosilicate (TEOS, 99+%) were purchased from Sigma-Aldrich (Singapore) and used as received. Carboxyethylsilanetriol (CEST), disodium salt (25% in water), was purchased from Gelest, Inc. (Morrisville, PA) and used without further purification. Rabbit anti-HSA antibody (labeled with and without Alexa fluor-647), mouse anti-HSA antibody, and HSA protein were procured from Abcam (Singapore). Mouse anti-hCRP antibody was purchased from R&D systems, Singapore, and hCRP was purchased from Sigma-Aldrich, Singapore. ChonBlock™ blocking buffer was procured from Chondrex, Inc. (Redmond, WA). *N*-(3-Dimethylaminopropyl)-*N*′-ethylcarbodiimide hydrochloride (EDC) and *N*-Hydroxysulfosuccinimide sodium salt (NHS) (≥ 98%) were obtained from Sigma-Aldrich, Singapore, and used without purification. Centrifugal filer units of size 5 μm were purchased from Millipore, Singapore.

### Multicolor UCNPs Synthesis and Surface Modification

Multicolor UCNPs were synthesized by hydro(solvo) thermal method as reported previously by our group [33]. β-NaYF_4_ was taken as the host lattice and doped with Yb^3+^ acting as sensitizer ion and Er^3+^ as activator ions to prepare UCNPs. NaYF_4_:50%Yb1%Er and NaYF_4_:30%Yb2%Er18%Lu UCNPs emitting red and green colors, respectively, were prepared by using different concentrations of Yb^3+^ and Er^3+^ ions. To prepare NaYF_4_:30%Yb0.5%Tm UCNPs emitting blue color, Tm^3+^ was used as activators.

For surface modification, UCNPs were grafted with carboxyl groups that involved an intermediate silica coating. Silica coating was achieved by previously reported reverse microemulsion method [33] followed by carboxyl groups modification via silane coupling method [34].

### Fabrication of Microfluidic Device

A silicon master was coated with a layer of SU8-2050 photoresist using a spin coater (Cee, Brewer science), while a positive silicon master was synthesized by using a mask aligner (Karl-SUSS Micro Tec.) equipped with UV-light exposure. Using this master, a microfluidic device was prepared by PDMS via soft lithography. After punching the input and output holes, the as-formed PDMS device was bonded to a glass slide substrate using oxygen plasma treatment.

### Synthesis of UCNPs-Encoded Microbeads

The precursor of microbeads consisting of carboxyl-modified NaYF_4_:50%Yb1%Er UCNPs (40%), PEGDA monomer (20%), photoinitiator (5%), and ethanol solution (20%) was prepared. Briefly, water solution of carboxyl-modified UCNPs was mixed with PEGDA under vortexing for 30 min. Photoinitiator was dissolved in ethanol (1:4 weight/volume ratio) under vortexing and sonication for 5 min. PEGDA-UCNPs solution was then mixed with the photoinitiator-ethanol solution and further vortexed for 10 min. As-prepared microbeads precursor solution serving as the water phase was filled into a glass syringe (Hamilton). An oil phase containing 2% of Pico-Surf™ 2 surfactant in HFE-7500 oil was filled into a 1-mL glass syringe. The oil and water phase solutions were driven by syringe pumps (Chemyx Inc.) and co-flown through the microfluidic device at flow rates of 4 and 1 μL min^−1^, respectively. The formed droplets were collected in a plastic tube and crosslinked under 365-nm UV exposure for 1 min. Thereafter, crosslinked microbeads were washed three times each with isopropyl alcohol (IPA) and DI water prior to further use. By changing the flow rates of oil phase from 2 to 20 μL min^−1^, microbeads of different sizes were prepared. Additionally, multicolor microbeads were prepared by mixing carboxyl-modified NaYF_4_:50%Yb1%Er and NaYF_4_:30%Yb2%Er18%Lu UCNPs at different volume ratios of 1:0, 0.67:0.33, 0.5:0.5, 0.33:0.67, and 0:1.

### Surface Modification of Encoded Microbeads

UCNPs-encoded microbeads (~ 12,000/100 μL) in water solution were suspended in a glass bottle under magnetic stirring at 350 rpm. This was followed by sequential addition of 33% ammonia and 8 μL of TEOS. After 1 day of stirring, 16 μL of CEST was added and further stirred for another day. Next, the solution of carboxyl-modified microbeads was collected in a 1.5 mL low-adhesion tube (Bio-plastics Singapore) and pelleted down under centrifugation at 6000 rpm for 2 min. The microbeads were washed with 1 mL DI water for three times prior to further use and characterized for quantification and functionality of carboxyl groups post-modification. The carboxyl groups on surface-modified microbeads were quantified by BCG assay, following the manufacturer’s protocol. To prepare the standard curve, known concentrations of CEST were taken. Further, the functionality of carboxyl groups on the surface-modified UCNPs-encoded microbeads was analyzed by EDC/NHS-based covalent coupling of Alexa Fluor 647-labeled goat anti-HSA antibody. Briefly, 30 µL (~ 3600) of carboxyl-modified microbeads were incubated with water solutions of 0.125 mmol NHS and 0.05 mmol EDC under vortexing at 1400 rpm for 30 min at 4 °C. The microbeads with activated carboxyl groups on their surface were washed thrice with DI water, resuspended in DI water and incubated with Alexa Fluor 647-labeled goat anti-HSA antibody under vortexing at 1400 rpm for 3 h at 4 °C. After antibody conjugation, the microbeads were washed thrice with DI water prior to further characterizations.

The amount of carboxyl groups imparted on the surface of microbeads was estimated based on Bromocresol Green (BCG) assay kit from Sigma-Aldrich, Singapore. The manual provided with the manufacturer’s kit was followed to perform the BCG assay; however, the standard chosen was the known concentrations of carboxyl groups in water solution of CEST.

### Preparation of UCNPs-Labeled Reporter Antibody

Mouse anti-HSA reporter antibody was conjugated with different concentrations of carboxyl-modified NaYF_4_:30%Yb0.5%Tm UCNPs (0.25–2 mg mL^−1^). Briefly, carboxyl groups of UCNPs were first activated by incubation with 0.2 mmol NHS and 0.08 mmol EDC under vortexing at 1650 rpm for 30 min at 4 °C. After this, the UCNPs were pelleted down and washed three times with DI water under centrifugation at 13,000 rpm for 15 min and resuspended in DI water by pipette mixing and sonicated for 30 s. Next, the UCNPs were incubated with 0.25 mg mL^−1^ of mouse anti-HSA antibody at 1650 rpm for 3 h at 4 °C. After which, UCNPs-anti-HSA antibody was pelleted down and washed thrice with DI water by centrifugation at 13,000 rpm for 15 min prior to further use.

### UCNPs-Encoded Microbead-Based Multiplexed Sandwich Immunoassays

First, probe antibody goat anti-HSA polyclonal antibody (Abcam, Singapore) was immobilized on carboxyl-modified UCNPs-encoded microbeads by EDC/NHS carbodiimide covalent coupling, as described above. Next, 100 µL of 0.1% ChonBlock™ blocking buffer was incubated with the probe antibody-conjugated microbeads with vortexing at 1400 rpm for 2 h at room temperature. Afterward, target HSA protein was mixed with the microbeads for 1 h. The microbeads were washed thrice with DI water at 6000 rpm for 2 min at room temperature. Finally, the microbeads were incubated with NaYF_4_:30%Yb0.5%Tm UCNPs-labeled mouse anti-HSA monoclonal reporter antibody for 30 min to detect the proteins bound on the probe antibody-coated microbeads. Prior to imaging, the microbeads system was washed using filtration through 5 µm centrifuge filter at 3000 rpm for 2 min. Multiplexed detection was demonstrated by conjugating sheep anti-hCRP polyclonal probe antibody on the surface of NaYF_4_:18%Yb2%Er30%Lu UCNPs-encoded microbeads followed by target hCRP binding and detection by NaYF_4_:30%Yb0.5%Tm UCNPs-labeled mouse monoclonal anti-hCRP reporter antibody.

### Characterizations

Fluorescence spectra of all the UCNPs used for encoding the microbeads and labeling of the reporter antibody were recorded using SpectroPro 2150i spectrophotometer (Roper Scientific Acton Research, MA) equipped with a 980 nm NIR continuous laser (Photonitech (Asia) Pte. Ltd., Singapore). The dynamic light scattering (DLS) hydrodynamic diameter, size distribution, and zeta potential (surface charge) were measured by Malvern Zetasizer nanoseries (Malvern Instruments Ltd., UK). Transmission electron microscopy (TEM, JEOL 2010F TEM, Jeol Ltd., Tokyo, Japan) was used to measure the size and distribution of all the UCNPs. The sizes of nanoparticles were calculated using ImageJ (version 1.50i). Fluorescent images of nanoparticles without any filter were captured by a Canon 550D camera (Canon Inc., Tokyo, Japan) under 980 nm excitation from a continuous wave laser. Fourier transform infrared spectroscopy (FTIR) to confirm the surface modification of UCNPs was performed using IR Prestige-21 FTIR (Shimadzu Corporation, Japan). FTIR samples were prepared by grinding the nanoparticles (control and modified) with potassium bromide (KBr)-disk pellet method, and the FTIR spectra of the samples were drawn in transmittance mode.

Zeiss optical microscope with a high-speed camera was used to monitor and record the droplet synthesis in the microfluidic device. The bright field and fluorescent micrographs of the crosslinked microbeads and the sandwich assay established on the encoded microbeads were captured using a CytoViva microscope (Nikon 80i Fluorescence Microscope, Nikon, Tokyo, Japan) equipped with continuous wave 980 nm laser excitation source (Changchun New Industries Optoelectronics Tech. Co. Ltd). The corrected fluorescence of the encoded microbeads before and during sandwich assay was calculated using ImageJ by the following formula:

1 $${\text{Corrected}}\;{\text{total}}\;{\text{fluorescence}}\, = \,{\text{Integrated}}\;{\text{density}}\,{-}\, ( {\text{Area}}\;{\text{of}}\;{\text{selected}}\;{\text{beads}}\, \times \,{\text{Mean}}\;{\text{fluorescence}}\;{\text{of}}\;{\text{background}}\;{\text{readings)}}$$

## Results and Discussion

### UCNPS Synthesis and Surface Modification for Encoding of Microbeads

To prepare the UCNPs, NaYF_4_ was chosen as the host lattice as it ensures homogeneous accommodation of dopants (sensitizers and activator ions) as well as maximum radiative emissions and minimum lattice stress and non-radiative energy owing to its low phonon energy. Based on the type and concentrations of the dopant ions, three different UCNPs emitting red, green, and blue colors were prepared in this study.

**Fig. 2:**
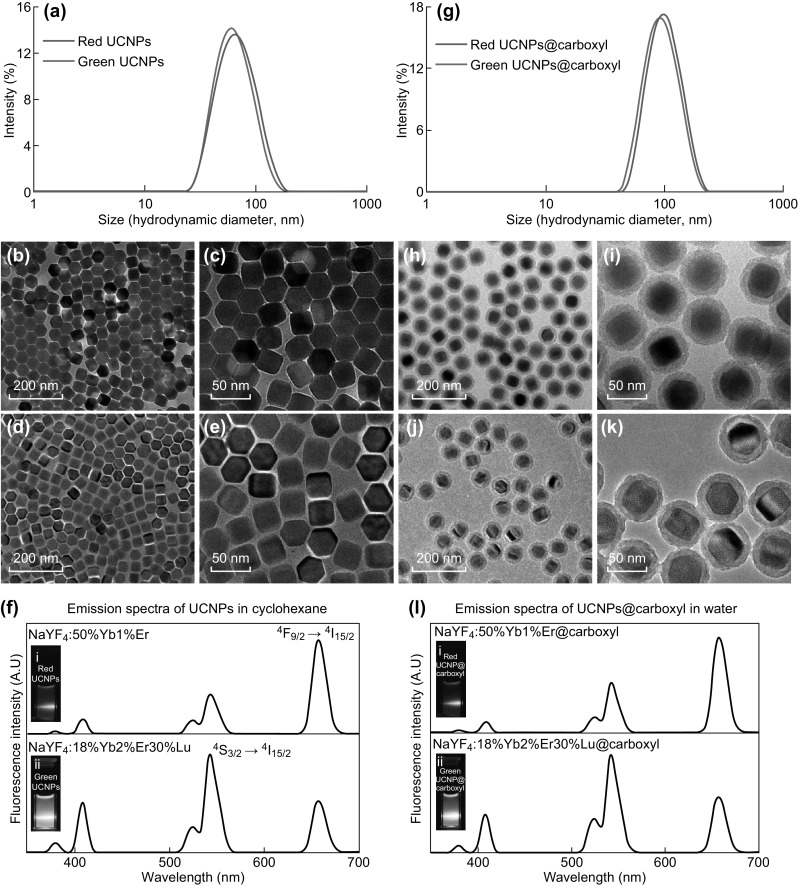
**a** Hydrodynamic size and size distribution of NaYF_4_:50%Yb1%Er (red) and NaYF_4_:18%Yb2%Er30%Lu (green) UCNPs in dispersed cyclohexane. TEM images of **b, c** NaYF_4_:50%Yb1%Er and **d, e** NaYF_4_:18%Yb2%Er30%Lu UCNPs dispersed in cyclohexane. **f** Emission fluorescence spectra of NaYF_4_:50%Yb1%Er (top) and NaYF_4_:18%Yb2%Er30%Lu (bottom) UCNPs insets (i, ii) are corresponding camera pictures. **g** Hydrodynamic size and size distribution of carboxyl-modified NaYF_4_:50%Yb1%Er (red) and NaYF_4_:18%Yb2%Er30%Lu (green) UCNPs dispersed in water. TEM images of carboxyl-modified **h, i** NaYF_4_:50%Yb1%Er and **j, k** NaYF_4_:18%Yb2%Er30%Lu UCNPs dispersed in water. **l** Emission fluorescence spectra of carboxyl-modified NaYF_4_:50%Yb1%Er (top) and NaYF_4_:18%Yb2%Er30%Lu (bottom) UCNPs insets (i, ii) are corresponding camera pictures. (Color figure online)

For encoding of microbeads, Yb^3+^ and Er^3+^ dopants were selected as sensitizers and activator ions, respectively. By varying the concentrations of these dopants, NaYF_4_:50%Yb1%Er UCNPs emitting red color and NaYF_4_:18%Yb2%Er30%Lu UCNPs emitting green color were prepared. The DLS data of the UCNPs dispersed in cyclohexane showed a single peak with narrow size distribution, thereby signifying absence of aggregation (Fig. 2a). The hydrodynamic diameter was found to be 68.93 ± 1.87 and 67.35 ± 0.21 nm for NaYF_4_:50%Yb1%Er and NaYF_4_:18%Yb2%Er30%Lu UCNPs, respectively. TEM images (Fig. 2b–e) showed that both the UCNPs have the uniform shape and size with hexagonal geometry. The size of NaYF_4_:50%Yb1%Er and NaYF_4_:18%Yb2%Er30%Lu UCNPs is (36.28 ± 0.99) (height, nm) × (32.28 ± 0.55) (width, nm) and (35.87 ± 1.68) (height, nm) × (31.81 ± 0.57) (width, nm), respectively. From fluorescence spectra (Fig. 2f), although the two UCNPs had similar emission peaks, their intensities are different at different wavelengths. As shown in Fig. 2f, NaYF_4_:50%Yb1%Er UCNPs displays its strongest peak at 657 nm wavelength corresponding to ^4^F_9/2_ → ^4^I_15/2_ transition, thereby resulting in an overall red color fluorescence, as shown in the camera pictures in the inset of Fig. 2f. On the contrary, the strongest peak for NaYF_4_:18%Yb2%Er30%Lu UCNPs emitted at 543 nm wavelength is attributed to ^4^S_3/2_ → ^4^I_15/2_ transition, which in turn led to its overall green color fluorescence. Of note, the addition of Lu^3+^ led to an increase in size of the NaYF_4_:18%Yb2%Er30%Lu UCNPs. This was deliberately done to produce the similar size particles as that of the NaYF_4_:50%Yb1%Er UCNPs in order to achieve uniform co-encapsulation and subsequent fluorescence from the multicolor microbeads. The doped Lu^3+^, however, does not have any overall effect on the emission of UCNPs [35].

In order to impart hydrophilicity on these UCNPs, they were first surface modified prior to be used. To achieve this, a layer of silica was coated on the UCNPs, followed by their surface modification with carboxyl groups. The presence of carboxyl groups provides extra stability for their uniform dispersion inside the microbeads during encapsulation, as compared to encapsulation of silica-coated UCNPs alone (without carboxyl groups) that showed aggregation post encapsulation. As observed from DLS data (Fig. 2g), both the carboxyl-modified NaYF_4_:50%Yb1%Er and NaYF_4_:18%Yb2%Er30%Lu UCNPs have a single peak with narrow size distribution, implying absence of aggregation when dispersed in water. The average hydrodynamic size of the NaYF_4_:50%Yb 1%Er@carboxyl and NaYF_4_:18%Yb2%Er30%Lu@carboxyl UCNPs is 94.6 ± 0.03 and 89.83 ± 0.98 nm, respectively. The TEM images showed that surface modification did not affect the shape of the UCNPs. The average size of the NaYF_4_:50%Yb1%Er@carboxyl and NaYF_4_:18%Yb2%Er30%Lu@carboxyl UCNPs is (71.4 ± 0.58) (height, nm) × (65.93 ± 1.55) (width, nm), while that of NaYF_4_:18%Yb2%Er30%Lu@COOH UCNPs is (69.22 ± 1.00 (height, nm) × (65.73 ± 0.22) (width, nm), as shown in Fig. 2h–k. For both UCNPs, their characteristic emission peaks are preserved post-carboxyl modification, as shown in Fig. 2l.

To further confirm the successful surface modification, zeta potential of NaYF_4_:50%Yb1%Er UCNPs modified with silica and carboxyl groups was recorded, with the latter possessing a more negative charge (− 53.63 ± 2.06 mV) as compared to the former (− 43.33 ± 1.55 mV). Additionally, FTIR spectra were recorded for the unmodified NaYF_4_:50%Yb1%Er UCNPs and the ones modified with silica and carboxyl groups (Fig. S1f). The transmission band peaks of unmodified UCNPs capped with OA groups are at 2930, 2854, and 1705 cm^−1^ that are attributed to the asymmetric and symmetric stretching vibrations of methylene (–CH_2_) groups in the long alkyl chain, and stretching vibration of C=O in OA. Band peaks observed at 1553 and 1458 cm^−1^ corresponding to the asymmetric and symmetric vibrations of carboxylate anions in OA, respectively. The silica-coated UCNPs showed transmission band peaks at 1083, 954, and 800 cm^−1^, which could be assigned to Si–O–Si stretching, Si–OH stretching, and Si–O–Si bending, respectively. They are still preserved even after carboxyl modification of UCNPs. Notably, carboxyl-modified UCNPs had additional peaks at 1553 and 1458 cm^−1^ corresponding to carboxylate anions, which in turn signified successful carboxyl group modification on the UCNPs.

### Multicolor UCNPs-Encoded Microbeads Synthesis via Droplet Microfluidics

**Fig. 3:**
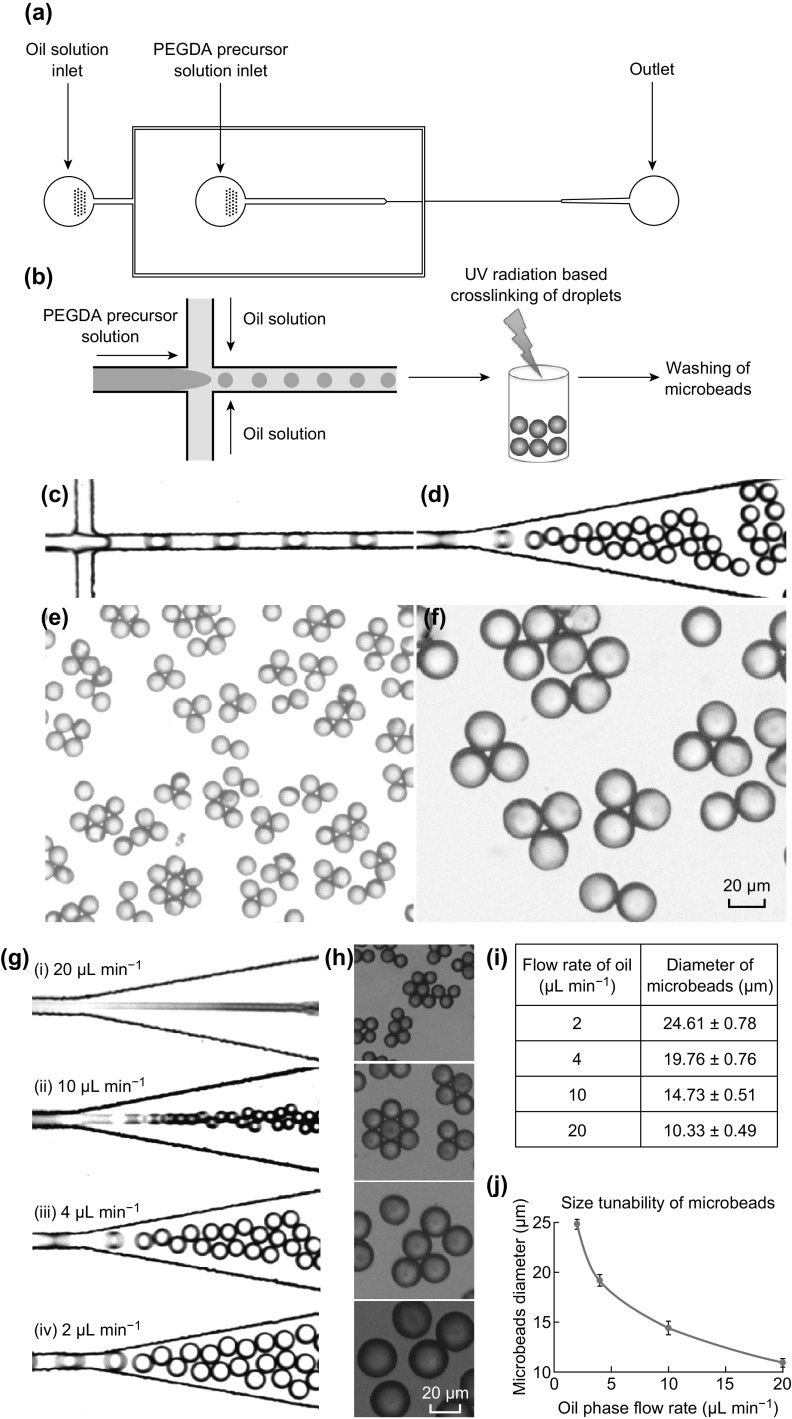
Droplet microfluidic-assisted synthesis of microbeads. **a** AutoCAD design of the droplet microfluidic device. **b** Schema showing mechanism of droplet microfluidic-based microbeads synthesis. **c, d** Bright field microscopy images showing the production of hydrogel using microfluidic device and **e, f** bright field microscopy images of microbeads formed after crosslinking the hydrogel at 10× and 20× magnifications. **g** Formation of hydrogel in the microfluidic channel at different flow rates of oil, and **h** corresponding bright field microscopy images (at 40×, exposure = 10 ms, gain = 4×) of microbeads formed. **i** Diameters of microbeads formed under the different oil flow rates, and **j** corresponding graph to show the relationship with different oil flow rates (data represent mean ± SD; *n* = 50 microbeads)

Microfluidic device was designed to prepare the microbeads consisted of a cross-junction and three holes: one inlet each for water and oil phase, the other outlet for collecting the hydrogel formed (Fig. 3a). At the cross-junction, incoming oil stream from the sides cuts the water stream to make the hydrogel droplets. These droplets were collected at the outlet and were subjected to photocrosslinking by UV to obtain the solidified microbeads (Fig. 3b). As shown in Fig. 3c–f, the formed microbeads are uniform size and shape. The size of these microbeads can be controlled by manipulating the flow rate of oil phase, flow rate of water phase, and the channel size at the cross-junction. Here, we tried to tune the microbeads size by varying the flow rate of oil phase from 2 to 20 µL min^−1^, while the water phase flow rate fixed at 1 μL min^−1^ (Fig. 3g, h). The size of the microbeads obtained is ranging from 11 to 25 µm, thereby signifying that the size of the microbeads is inversely proportional to the flow rate of oil phase (Fig. 3i, j).

The microfluidic device was utilized to encapsulate UCNPs inside the microbeads. This was achieved by dispersing the surface-modified UCNPs in a water solution of bead precursor prior to the droplet formation. Initially, silica-coated NaYF_4_:50%Yb1%Er UCNPs were used for encapsulation inside the microbeads. However, the silica-coated UCNPs showed apparent aggregation inside the microbeads upon encapsulation, as observed by their non-uniform fluorescence intensity (Fig. S2a, c). On the contrary, encoded microbeads obtained using carboxyl-modified NaYF_4_:50%Yb1%Er UCNPs display uniform fluorescence, as shown in Fig. S2b, d. A probable reason could be that the carboxyl groups impart a more pronounced negative charge on the UCNPs as compared to that conferred just by silica coating, which is essential for maintaining the required repulsion between the UCNPs for better stability inside the microbeads.

**Fig. 4:**
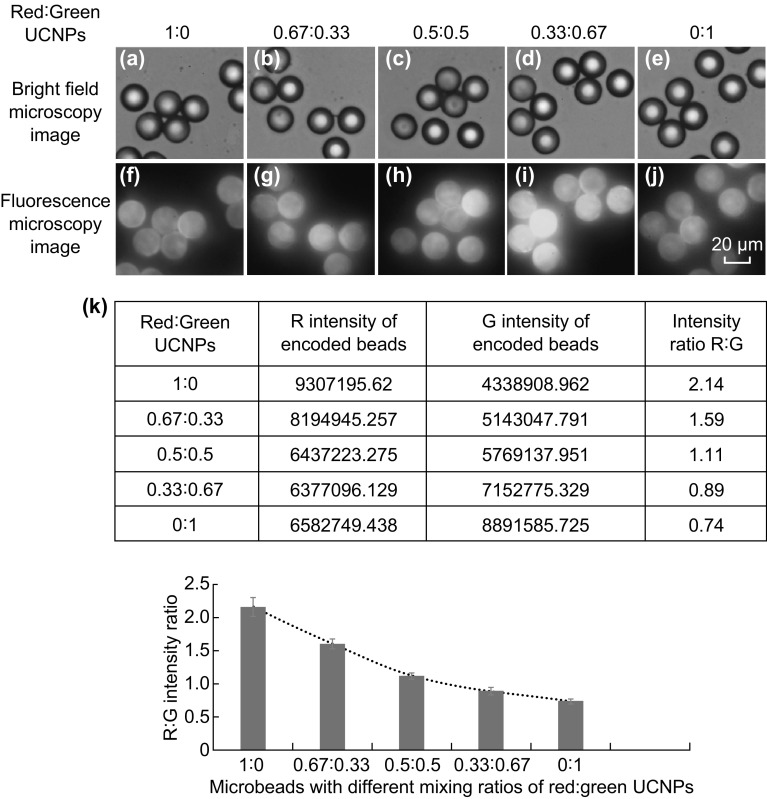
Bright field microscopy images and their corresponding fluorescent microscopy images (at 40× magnification) of microbeads encoded with carboxyl-modified NaYF_4_:50%Yb,1%Er and NaYF_4_:18%Yb2%Er30%Lu UCNPs at ratios of **a, f** 1:0, **b, g** 0.67:0.33, **c, h** 0.5:0.5, **d, i** 0.33:0.67, and **e, j** 0:1, **k** Ratio of red and green intensity calculated from each microbeads (top) and the corresponding graph (bottom) (data represent mean ± SD; *n* = 20 microbeads). (Color figure online)

Based on this finding, carboxyl-modified UCNPs were chosen for encapsulation inside the microbeads in all subsequent studies. In order to get the optimum concentration of UCNPs, fluorescence spectra of NaYF_4_:50%Yb1%Er UCNPs at different concentrations (0.25–4 mg mL^−1^) were recorded (Fig. S2e). The area under each spectrum was calculated using OriginPro, and the emission intensity was normalized to the lowest concentration of UCNPs used (0.25 mg mL^−1^). The normalized fluorescence intensity was plotted against the different concentrations of UCNPs, and the fluorescence is directly proportional to the concentrations of UCNPs in a linear fashion until 2 mg mL^−1^. At higher concentration beyond 2 mg mL^−1^, the fluorescence intensity no longer follows a linear relationship, which could be due to the diffraction of light at higher concentration of UCNPs in solution (Fig. S2e, f). Thus, 2 mg mL^−1^ of UCNPs was selected as the concentration to encode the microbeads, and five different multicolor microbeads were prepared by encapsulating carboxyl-modified NaYF_4_:50%Yb1%Er and NaYF_4_:18%Yb2%Er30%Lu UCNPs at different volume ratios of 1:0, 0.67:0.33, 0.5:0.5, 0.33:0.67, and 0:1, as shown in Fig. 4a–j.

To assess the unique identity of the color codes, the fluorescence intensity of each microbead was quantitated by ImageJ using Eq. 1. The corrected total fluorescence intensity corresponding to red (R) and green (G) emissions was calculated, and the ratio of R/G values was plotted against their specific ratios used for encoding the microbeads (Fig. 4k). The distinct R/G values for each microbead are inversely proportional to the different ratios of carboxyl-modified NaYF_4_:50%Yb1%Er and NaYF_4_:18%Yb2%Er30%Lu UCNPs, with increasing amount of the latter. It was inferred that the five encoded microbeads were distinct qualitatively (microscopy images) as well as quantitatively (fluorescence intensity measured by ImageJ) and hence deemed suitable for carrying out multiplexed detection.

### Surface Modification of Encoded Microbeads

Microbeads require functional groups on their surface for immobilization of probe antibodies to ensure specific capture of the target protein. Initially, we attempted to co-polymerize acrylic acid (AA) with PEGDA monomers to render carboxyl groups onto the microbeads surfaces, as reported earlier [36]. However, in the presence of AA, the fluorescence of encoded microbeads is significantly reduced. Another reported method involved coating polydopamine (PDA) on the surface of encoded microbeads [31]. But PDA quenches the emitted fluorescence, thereby restricting their use in fluorescence detection [37]. Additionally, the immobilization of probe antibodies on the PDA-PEGDA microbeads was done by physical adsorption, which might prove challenging to maintain the stability of immobilized probe antibodies on the microbeads. In view of these, we have hereby modified the NaYF_4_:50%Yb1%Er@carboxyl UCNPs-encoded PEGDA microbeads surface with carboxyl groups, following their coating with a layer of silica.

It was important to quantify the amount of carboxyl groups on the microbeads surface, the functionality of these carboxyl groups, as well as investigate the morphological (size and shape) and fluorescence stability of the encoded microbeads. BCG assay was chosen to estimate the amount of carboxyl groups on the microbeads surface [38]. Based on the standard curve plotted using known carboxyl concentrations in standard solutions of CEST (Fig. S3a), the amount of carboxyl groups on the microbeads surface was approximately estimated to be 0.17 M/5 µL of microbeads sample or 6.78 × 10^10^ mol of carboxyl groups/microbead.

To investigate the functionality of carboxyl groups on the surface of encoded microbeads, Alexa Fluor 647-labeled antibodies were conjugated to it via EDC/NHS chemistry. Based on bright field and fluorescence microscopy images, it was found that the size, shape, and UCNPs fluorescence of the encoded microbeads were unaffected for both the control microbeads (without antibodies) and microbeads with antibodies conjugated (Fig. S3b–g). In the case of microbeads with antibodies conjugated, however, an additional fluorescence corresponding to the emission of Alexa Fluor 647 dye was clearly observed, which was absent in control microbeads.

Following the carboxyl modification, the UCNPs-encoded microbeads were subjected to vigorous stirring (at 1400 rpm, with imaging done at regular intervals for up to 12 h) and centrifugation to assess their morphological and fluorescence stability. Under both these conditions, the encoded microbeads did not show any apparent change in shape, size, and fluorescence compared to the control microbeads without any treatment (Fig. S3h–m). For those subjected to stirring, the fluorescence of the microbeads was imaged and quantified using ImageJ at regular time intervals over a 12-h long study. This duration was selected to conduct the stability study based on the time required to perform one cycle of bio-detection (around 7–8 h inclusive of all the steps required in a sandwich immunoassay). As shown in Fig. S3n, after 12 h of stirring, the fluorescence intensity is still around 90% of the intensity of original microbeads, thereby suggesting that the encoded microbeads are stable after surface modification.

These studies confirmed that the surface of encoded microbeads was successfully modified with carboxyl groups and that the fluorescence of UCNPs encapsulated inside the microbeads as well as the functionality of carboxyl groups post-modification was preserved. Following this, an Alexa Fluor 647-labeled probe antibody against HSA target protein was conjugated on the encoded microbeads surface via EDC/NHS chemistry.

### UCNPs Synthesis and Surface Modification for Labeling Reporter Antibody

**Fig. 5:**
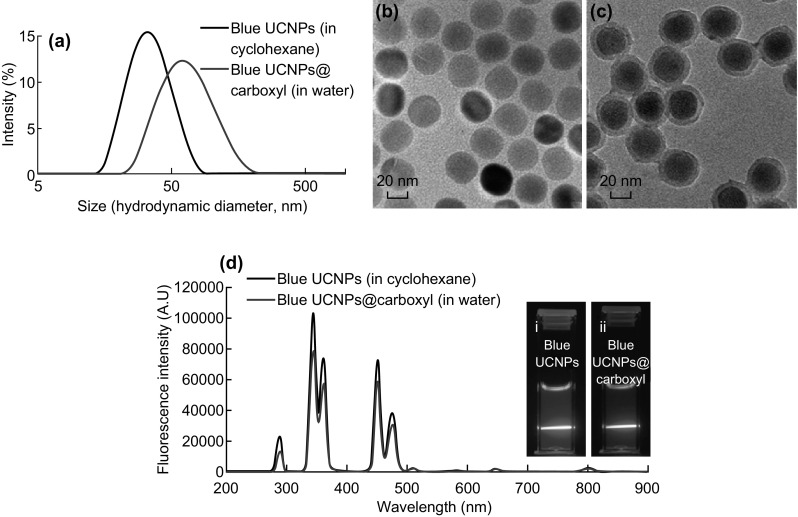
**a** Size distribution of NaYF_4_:30%Yb0.5%Tm UCNPs with and without carboxyl modification in cyclohexane and in water, respectively. TEM images of **b** NaYF_4_:30%Yb0.5%Tm UCNPs in cyclohexane, and **c** NaYF_4_:30%Yb0.5%Tm@carboxyl UCNPs in water. **d** Emission fluorescence spectra of NaYF_4_:30%Yb0.5%Tm UCNPs with and without surface modification in cyclohexane and in water, respectively, and insets are corresponding camera picture of (i) NaYF_4_:30%Yb0.5%Tm, and (ii) NaYF_4_:30%Yb0.5%Tm@carboxyl UCNPs

For labeling of reporter antibody, Yb^3+^ was maintained as the sensitizer ion, while the activator ion was switched to Tm^3+^ in order to prepare NaYF_4_:30%Yb0.5%Tm UCNPs emitting blue color. The hydrodynamic size distribution measurement of the resultant NaYF_4_:30%Yb0.5%Tm UCNPs showed uniform dispersion, as interpreted by the single peak displayed with narrow size distribution (Fig. 5a). The average hydrodynamic size of NaYF_4_:30%Yb0.5%Tm UCNPs is 31.99 ± 0.23 nm. As shown in TEM images (Fig. 5b), the blue UCNPs are spherical in shape with uniform size of 27.63 ± 0.81 nm. The fluorescence spectrum exhibited emission peaks at 288, 344, 360, 450, 475, 645, and 801 nm wavelength corresponding to ^1^I_6_ to ^3^H_6_, ^1^I_6_ to ^3^H_4_, ^1^D_2_ to ^3^H_6_, ^1^D_2_ to ^3^F_4_, ^1^G_4_ to ^3^H_6_, ^1^G_4_ to ^3^F_4_, and ^3^H_4_ to ^3^H_6_ transitions of Tm^3+^, respectively [39–41]. The overall blue fluorescence of NaYF_4_:30%Yb0.5%Tm UCNPs is shown in the camera picture in inset of Fig. 5d-i.

In the case of blue UCNPs, presence of carboxylic groups on their surface would provide functionality that is required for covalent conjugation of reporter antibody via their primary amine group using EDC/NHS chemistry. Carboxyl-modified NaYF_4_:30%Yb0.5%Tm UCNPs have an average hydrodynamic diameter of 60.13 ± 0.85 nm, without any aggregates (Fig. 5a). The surface-modified UCNPs are spherical in shape, with uniform size (35.99 ± 1.85 nm), size distribution, and coating, as observed in TEM images (Fig. 5c). Furthermore, carboxyl modification did not affect the characteristic emission spectra and overall blue color fluorescence of NaYF_4_:30%Yb0.5%Tm UCNPs, as shown in inset of Fig. 5d-ii.

### UCNPs-Conjugated Reporter Antibody Preparation

**Fig. 6:**
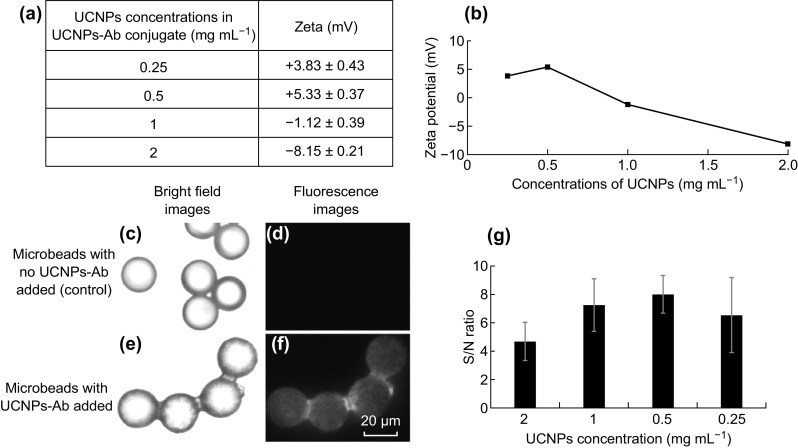
**a** Different surface charge of UCNPs-Ab conjugates at different concentrations of UCNPs, and **b** corresponding graph for surface charge versus UCNPs concentrations in UCNPs-Ab conjugate. **c, e** Bright field and **d, f** corresponding fluorescent microscopy images at 40× magnification of NaYF_4_:YbTm microbeads with and without UCNPs-Ab, and **g** corresponding S/N ratios of UCNPs-Ab at different concentrations of UCNPs used in the conjugation reaction (data represent mean ± SD; *n* = 20 microbeads)

Before developing the multiplexed bioassay, reporter antibody against the target protein was labeled with NaYF_4_:30%Yb0.5%Tm UCNPs to achieve an organic dye-free system with easy single wavelength excitation. Different concentrations of carboxyl-modified NaYF_4_:30%Yb0.5%Tm UCNPs ranging from 0.25 to 2 mg mL^−1^ were conjugated to a fixed amount of reporter antibody. The as-obtained UCNPs-antibody (UCNPs-Ab) with different concentrations of UCNPs was characterized for their surface charge to estimate the most optimum concentration of UCNPs suitable for conjugating the reporter antibody. Binding of antibodies to the UCNPs should lead to an increase in the overall charge of the nanoparticles such that they become less negative as compared to UCNPs without antibodies. As shown in Fig. 6a, b), it was found that the overall surface charge of UCNPs-Ab conjugate at higher concentrations of UCNPs is negative, suggesting that they are only partially covered with antibodies due to the excess amount of UCNPs present in the reaction mixture. UCNPs-Ab prepared at 0.5 mg mL^−1^ UCNPs concentration, however, acquired the most positive charge, which suggests for the optimum concentration of UCNPs to be used to achieve the highest amount of antibody conjugated.

The optimum UCNPs concentration was further examined by conjugating UCNPs-Ab to the microbeads surfaces. The fluorescent images of microbeads bound to UCNPs-Ab at different concentrations of UCNPs (Fig. 6c–f) were analyzed to obtain the fluorescence intensity (signal). Then, taking background noise into account, a ratio of signal/noise (S/N) was calculated and plotted against the different concentrations of UCNPs on the microbeads (Fig. 6g). The S/N ratio is highest in UCNPs-Ab sample prepared with 0.5 mg mL^−1^ concentration of UCNPs in the reaction mixture. This finding is in accordance with the data obtained for zeta potential. Thus, for subsequent studies, 0.5 mg mL^−1^ of blue UCNPs were chosen as the concentration to be used for labeling the reporter antibody. The optimized UCNPs-Ab conjugate prepared at 0.5 mg mL^−1^ of blue UCNPs was also characterized for DLS size (to estimate the average hydrodynamic size and size distribution), absorbance from 230 to 400 nm (to affirm the binding of antibody), and fluorescence emission spectra (to evaluate the characteristic peaks of UCNPs after conjugation of antibody). As shown in Fig. S4a, the DLS measurement showed a narrow peak for UCNPs-Ab conjugate, thereby representing lack of any aggregates. Notably, the overall hydrodynamic sizes of the conjugates are more than that of the control unconjugated UCNPs. This could be attributed to the retardation of Brownian diffusion rate of UCNPs after antibody conjugation, thereby leading to an increase in the overall hydrodynamic diameter [42, 43]. The absorbance spectra measured from 230 to 400 nm showed a small peak in the UCNPs-Ab conjugate that matches well with the characteristic peak of pure antibody, which proved the successful labeling of antibody with UCNPs (Fig. S4b). Moreover, the characteristic peaks of NaYF_4_:30%Yb0.5%Tm UCNPs are still preserved after conjugation to antibody, as shown in Fig. S4c.

### Multicolor UCNPs-Encoded Bead-Based Sandwich Immunoassay

**Fig. 7:**
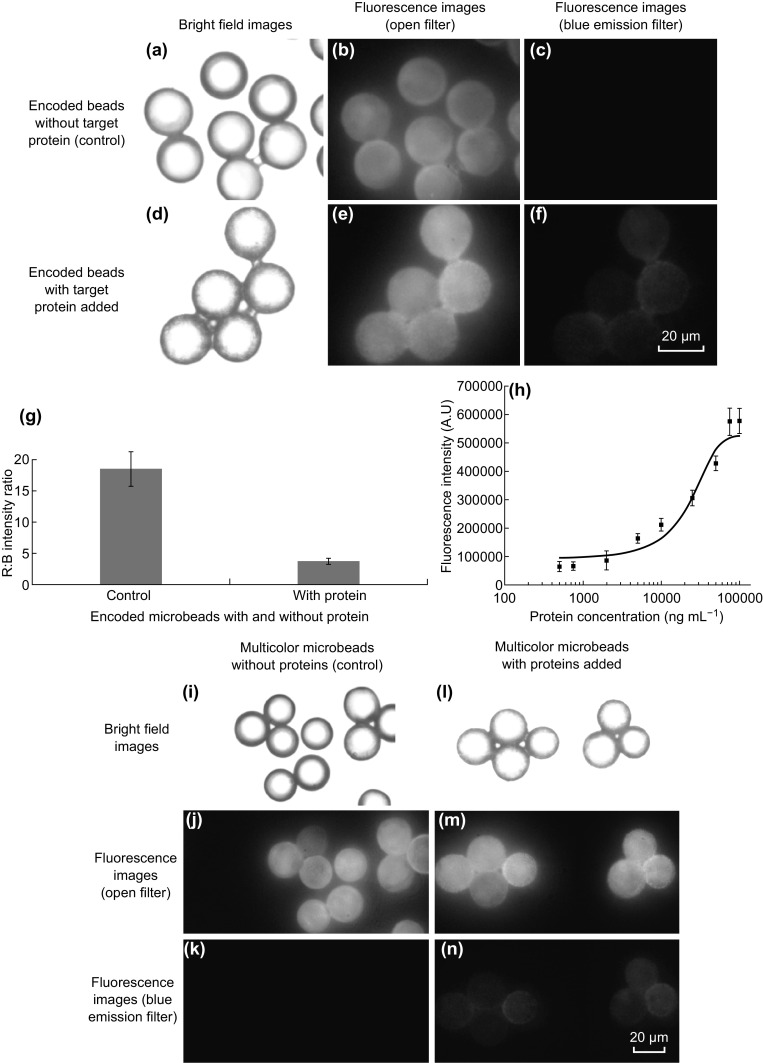
Bright field images and corresponding fluorescent microscopy images of open filter and filter for collecting blue emissions (at 40× magnification). **a, b, c** without (control) and **d, e, f** with target HSA protein added. **g** Plot of R/B intensity ratio for encoded microbeads with and without protein target added for detection (data represent mean ± SD; *n* = 20 microbeads). **h** Calibration curve for quantitative detection of target HSA protein (data represent mean ± SD; *n* = 20 microbeads). Multiplex detection of target proteins HSA and hCRP using NaYF_4_:50%Yb1%Er and NaYF_4_:18%Yb2%Er30%Lu UCNPs-encoded multicolor microbeads. Bright field images and corresponding fluorescent images of open filter and filter for collecting blue emissions for encoded microbead-based detection for **i, j, k** without (control) and **l, m, n** with target proteins added

The probe antibody-conjugated encoded microbeads were employed to detect model proteins to explore their potential in performing a multiplexed bioassay. To establish the assay, anti-HSA antibody was first coated on the surface of NaYF_4_:50%Yb1%Er@carboxyl UCNPs to detect HSA target protein (Fig. 7a–f). For excitation of UCNPs inside the microbeads and those used for labeling of reporter antibody, a single 980 nm continuous wave laser was employed. The emission signal from UCNPs inside the microbeads as well as from blue UCNPs labeling the reporter antibody was collected using open filter and blue emission filter, respectively. As shown in Fig. 7c, no blue fluorescence signal was observed for microbeads system without target HSA added. Whereas in the microbeads detection system with target HSA protein added, the binding of blue UCNPs-reporter antibody to the target protein bound on the microbeads was confirmed with the appearance of blue fluorescence, as displayed in Fig. 7f. Additionally, in the open filter mode, upconversion fluorescence emitted from the UCNPs-encoded microbeads showed a red-blue hybrid color when target HSA protein was added (Fig. 7e). This is yet again due to the binding of blue UCNP-reporter antibody to its target. No such observation was recorded in the microbeads system without target HSA protein added (Fig. 7b). It is worth to note that although the red UCNPs possessed a blue emission peak (as seen in Fig. 2), it is too weak to be captured by microscopy imaging and hence does not distinctly contribute toward the overall emission color detected. For both the microbeads systems with and without target HSA added, the images captured using open and blue emission filters were further analyzed by calculating the corrected fluorescence intensity of R and B using Eq. 1. The ratio of R/B is higher in the control microbeads system (no target HSA), which suggested that the blue UCNPs-reporter antibody was not attached. However, in the microbeads system with HSA target added, a significant reduction in the R/B was observed, further confirming the binding of blue UCNPs-reporter antibody to the target HSA on the microbeads (Fig. 7g). To evaluate the detection limit of this detection system, different concentrations of HSA proteins were added, and the standard curve was plotted between the fluorescence intensity of the microbeads and the respective protein concentrations (Fig. 7h). Based on the curve, the limit of detection (LOD) was calculated to be 7 µg mL^−1^, using the following formula:

2 $${\text{LOD}}\, = \,{{(3\, \times \,{\text{standard}}\;{\text{deviation}})} \mathord{\left/ {\vphantom {{(3\, \times \,{\text{standard}}\;{\text{deviation}})} {{\text{slope}}\;{\text{of}}\;{\text{the}}\;{\text{curve}}}}} \right. \kern-0pt} {{\text{slope}}\;{\text{of}}\;{\text{the}}\;{\text{curve}}}}$$

Following the above successful single-plex detection, the multiplexed assay was demonstrated on the multicolor microbeads for HSA and hCRP as target proteins. Microbeads encoded with R/G UCNPs in 1:0 and 0:1 ratios were utilized after conjugating anti-HSA and anti-hCRP antibodies against HSA and hCRP targets, respectively. Upon binding of the target proteins onto the encoded microbeads, monoclonal anti-HSA and monoclonal anti-hCRP detection antibodies labeled with blue UCNPs were added. Their bright field and fluorescence images were then captured by microscopy imaging (Fig. 7i–n). In control microbeads, with no target proteins added, no blue fluorescence was observed, implying that no attachment of any of the reporter antibodies took place (Fig. 7k). However, in microbeads with the two target proteins added, both types of microbeads targeting either against HSA or hCRP proteins (differentiated by their difference in overall fluorescence emitted in open filter) showed blue fluorescence, signifying the binding of both the blue UCNPs-reporter antibodies to their respective encoded microbead (Fig. 7n). These studies thereby demonstrated the multiplexed detection capability of the multicolor microbeads under a single wavelength excitation.

## Conclusion

In conclusion, we synthesized multicolor UCNPs emitting red and green colors for encoding PEGDA microbeads and UCNPs emitting blue color for labeling of reporter antibody and established multiplexed detection of model proteins on the multicolor microbeads. First, the UCNPs emitting red and green colors were modified with carboxyl groups to render them hydrophilic and allow uniform distribution inside the microbeads. Next, the droplet microfluidic device with cross-junction was fabricated to prepare the UCNPs-encoded microbeads. The microfluidics-assisted synthesis of UCNPs-encoded microbeads yielded five different color codes with uniform shape, size, and fluorescence. The size of the microbeads is tunable from 11 to 25 µm by varying the flow rate of oil phase. To achieve specific target detection, the surfaces of these encoded microbeads were modified with carboxyl groups for covalent conjugation to probe antibodies. For detection, reporter antibody was labeled with UCNPs emitting blue color, thereby enabling single wavelength excitation of the entire system. Ultimately, simultaneous detection of HSA and hCRP model proteins was demonstrated on UCNPs-encoded multicolor microbeads via sandwich assay in this proof-of-concept study. As shown previously, the LOD of the UCNPs-encoded bead-based detection system for HSA protein is 7 μg mL^−1^. For detecting smaller concentration of proteins, the LOD was improved by further optimizing the assay conditions to be at par with that of the currently available commercial protein detection methods such as microplate-based ELISA and Luminex’s xMAP technology. However, compared to the existing commercial methods, UCNPs-encoded bead-based detection approach offers several advantages. In comparison to ELISA, the microbeads used in our detection system provide a possibility to improve the protein detection efficiency due to their smaller surface area-to-volume ratio. Additionally, encoding the microbeads with multicolor UCNPs offers a flexible option of multiplexed protein detection. Furthermore, when compared to organic dyes-based Luminex’s xMAP technology, the use of UCNPs-based system enables the convenient single wavelength excitation and the detection is devoid of autofluorescence. Utilizing the benefits of UCNPs over traditionally used organic dyes for encoding and labeling, we envisage an improvement in the existing encoded microbeads-based multiplexed bioassay for a variety of applications.

## Electronic supplementary material

Below is the link to the electronic supplementary material.

Supplementary material 1 (PDF 867 kb)
