# Supplementary material for: Upconversion Nanoparticles-Encoded Hydrogel Microbeads-Based Multiplexed Protein Detection
Source: Nanomicro Lett. 2017 Dec 29;10(2):31. doi: 10.1007/s40820-017-0184-y (PMC6199079; doi:10.1007/s40820-017-0184-y)
Supplement: Supplementary file 1 — Supplementary material 1 (PDF 867 kb) [file 40820_2017_184_MOESM1_ESM.pdf]

Supporting Informaiton for

Upconversion Nanoparticles Encoded Hydrogel Microbeads-Based Multiplexed Protein Detection

Swati Shikha[1], Xiang Zheng[1, 2], Yong Zhang[1, 2, *]

[1]Department of Biomedical Engineering, Faculty of Engineering, 4 Engineering Drive 3, Block E4 #04-08, National University of Singapore (NUS), Singapore 117583

[2]NUS Graduate School for Integrative Sciences and Engineering, Centre for Life Sciences (CeLS), 05-01 28 Medical Drive, Singapore 117456

*Corresponding author. E-mail: biezy@nus.edu.sg

Figures

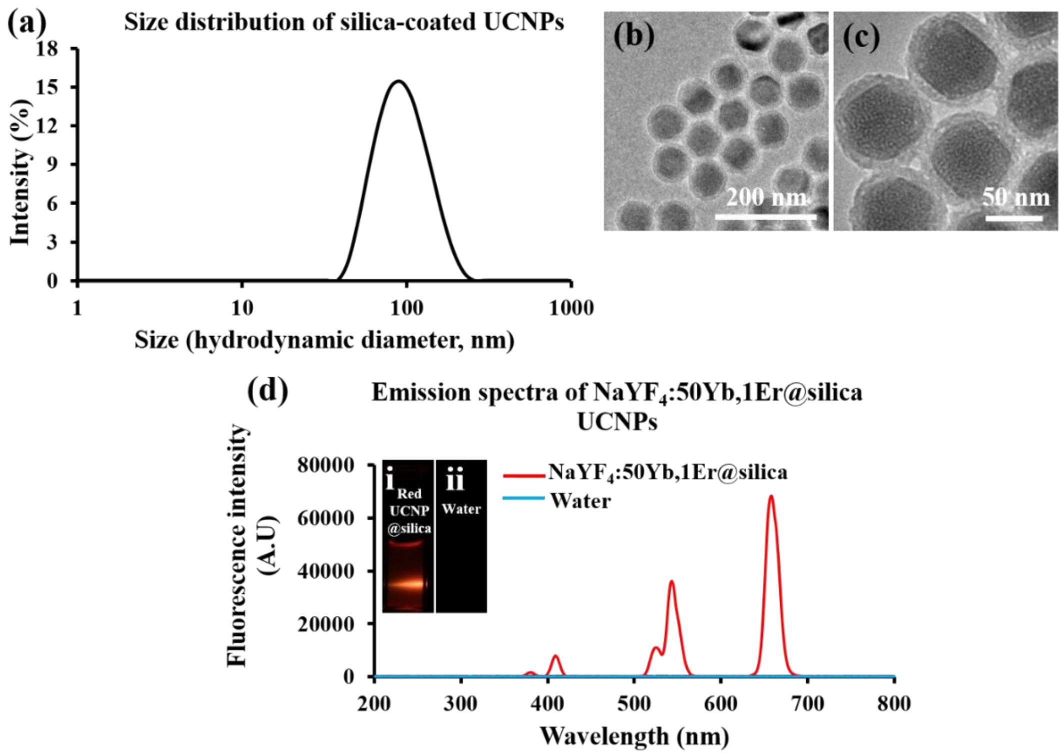

(e)

Sample	Zeta potential (mV)
Silica-coated UCNPs	-43.33 ± 1.55
Carboxyl-modified UCNPs	-53.63 ± 2.06

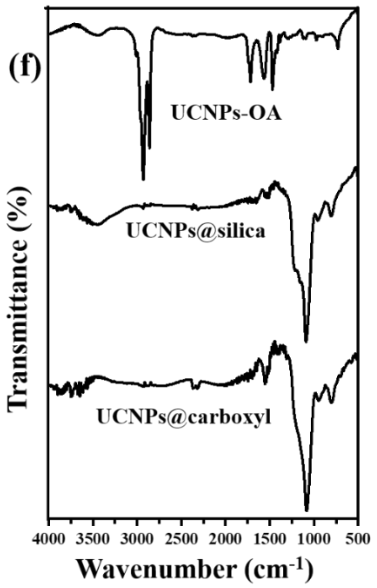

Fig. S1 Surface modification of UCNPs **a** DLS size distrubution, **b, c** TEM images at different magnifications, and **d** fluorescence emission spectra of silica coated UCNPs. **e** Zeta potential of silica-coated and carboxyl-modified UCNPs. **f** FTIR spectra of UCNPs before and after surface modification

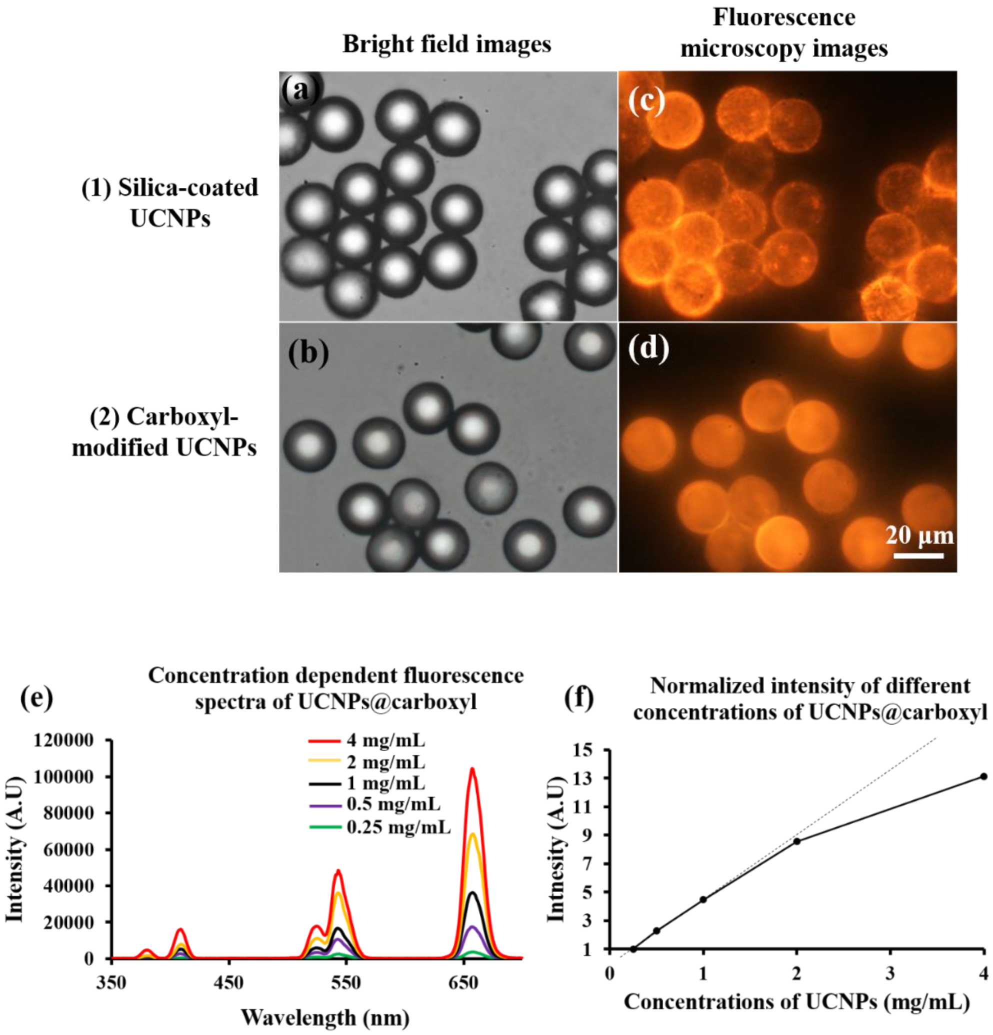

Fig. S2 Single color UCNPs encoded microbeads synthesis. **a, b** Bright field and **c, d** corresponding fluorescence microscopy images of microbeads encoded with silica-coated (Panel 1), and carboxyl-modified $NaYF_4$:50%Yb1%Er UCNPs (Panel 2). **e** Relationship between fluorescence intensity and concentration of carboxyl-modified $NaYF_4$:50%Yb1%Er UCNPs in water upon excitation under 980 nm NIR laser, and **f** their corresponding normalized fluorescence intensity

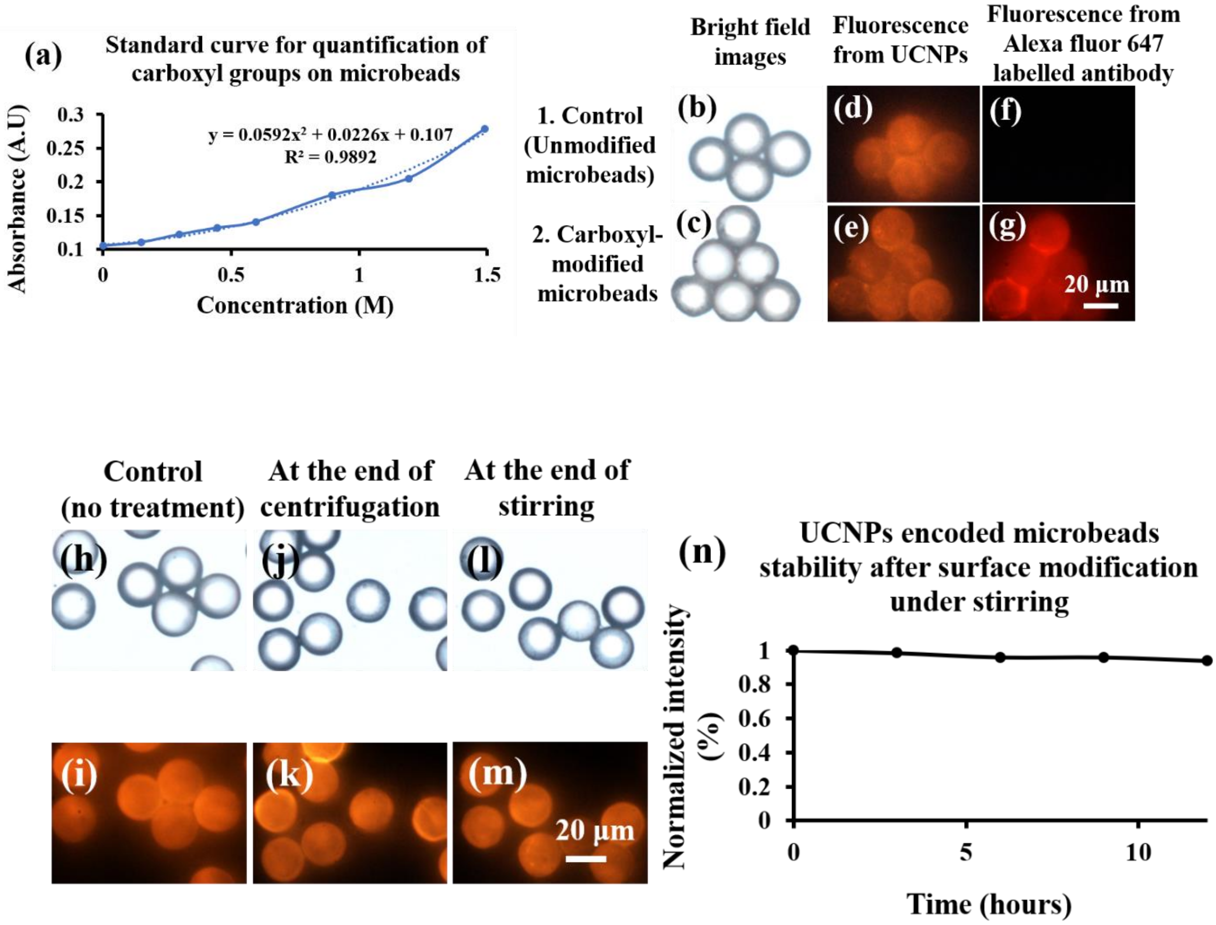

Fig. S3 Surface modification of UCNPs encoded microbeads. **a** Standard curve for BCG assay to quantify carboxyl groups on the microbeads surface (absorbance taken at 562 nm) **b, c** Bright-field and **d, e** corresponding fluorescence microscopy images showing UCNPs encapsulated inside the microbeads and **f, g** Alexa Fluor 647 labelled antibody to check the functionality of surface modified UCNPs encoded microbeads (at 40x magnification). Physical stability of surface modified UCNPs encoded microbeads. Bright-field and fluorescence microscopy images of **h, i** original microbeads without any treatment, **j, k** after subjecting to centrifugation, and **l, m** at the end of stirring experiments. **n** Normalized intensity of microbeads at different time points of stirring in accordance to assay conditions (n = 20 microbeads)

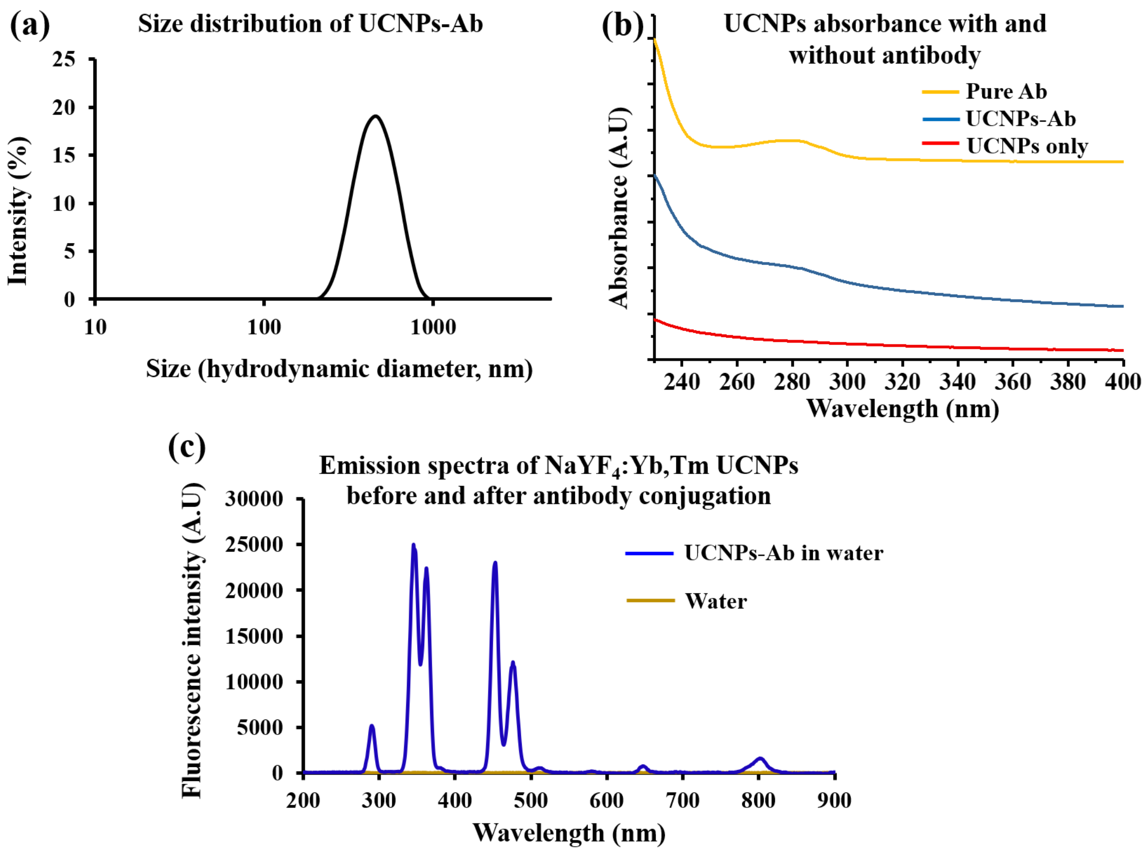

Fig. S4 Reporter antibody conjugated $NaYF_4$:30%Yb0.5%Tm UCNPs. **a** DLS showing average hydrodynamic diameter of $NaYF_4$:30%Yb0.5%Tm UCNPs conjugated to reporter antibody. **b** Absorbance spectrum of UCNPs, pure antibody and UCNPs conjugated antibody. **c** Emission fluorescence spectra of $NaYF_4$:30%Yb0.5%Tm UCNPs conjugated to reporter antibody in water and control (water only), under NIR laser excitation at 980 nm

Reference

[1] C. Yesildag, A. Tyushina, M. Lensen, Nano-contact transfer with gold nanoparticles on PEG hydrogels and using wrinkled PDMS-stamps. Polymers **9**(6), 199 (2017). https://doi.org/10.3390/polym9060199
